# Graphene as a Piezoresistive Material in Strain Sensing Applications

**DOI:** 10.3390/mi13010119

**Published:** 2022-01-12

**Authors:** Farid Sayar Irani, Ali Hosseinpour Shafaghi, Melih Can Tasdelen, Tugce Delipinar, Ceyda Elcin Kaya, Guney Guven Yapici, Murat Kaya Yapici

**Affiliations:** 1 Faculty of Engineering and Natural Sciences, Sabanci University, Istanbul TR 34956, Turkey; farid@sabanciuniv.edu (F.S.I.); alih@sabanciuniv.edu (A.H.S.); mctasdelen@sabanciuniv.edu (M.C.T.); tugcedelipinar@sabanciuniv.edu (T.D.); 2 Department of Electrical and Computer Engineering, University of Tulsa, Tulsa, OK 74104, USA; cek7838@utulsa.edu; 3 Department of Mechanical Engineering, Ozyegin University, Istanbul TR 34794, Turkey; guven.yapici@ozyegin.edu.tr; 4 Department of Electrical Engineering, University of Washington, Seattle, WA 98195, USA; 5 SUNUM Nanotechnology Research Center, Istanbul TR 34956, Turkey

**Keywords:** graphene, strain sensor, strain gauge, gauge factor, piezoresistance, piezoresistivity, MEMS, graphene transfer and integration

## Abstract

High accuracy measurement of mechanical strain is critical and broadly practiced in several application areas including structural health monitoring, industrial process control, manufacturing, avionics and the automotive industry, to name a few. Strain sensors, otherwise known as strain gauges, are fueled by various nanomaterials, among which graphene has attracted great interest in recent years, due to its unique electro-mechanical characteristics. Graphene shows not only exceptional physical properties but also has remarkable mechanical properties, such as piezoresistivity, which makes it a perfect candidate for strain sensing applications. In the present review, we provide an in-depth overview of the latest studies focusing on graphene and its strain sensing mechanism along with various applications. We start by providing a description of the fundamental properties, synthesis techniques and characterization methods of graphene, and then build forward to the discussion of numerous types of graphene-based strain sensors with side-by-side tabular comparison in terms of figures-of-merit, including strain range and sensitivity, otherwise referred to as the gauge factor. We demonstrate the material synthesis, device fabrication and integration challenges for researchers to achieve both wide strain range and high sensitivity in graphene-based strain sensors. Last of all, several applications of graphene-based strain sensors for different purposes are described. All in all, the evolutionary process of graphene-based strain sensors in recent years, as well as the upcoming challenges and future directions for emerging studies are highlighted.

## 1. Introduction

With the advent of the internet-of-things (IoT), smart, ubiquitous, pervasive sensing is rapidly gaining importance for providing reliable information at unprecedented sensitivity to enable new applications in consumer electronics [1,2,3], healthcare [4,5,6], manufacturing and structural monitoring [7,8], transportation [9,10], defense and surveillance [11,12,13]; as well as to fuel research in fundamental, applied and translational science [14]. Among the various physical measurands, the monitoring of strain finds use in numerous applications and industrial products where the fundamental detection principle relies on the change in electrical properties of the strain sensing element as a result of applied pressure or force. Strain sensors essentially rely on four fundamental sensing modalities which are capacitive, piezoelectric, piezoresistive and optical sensing [15]. Among these, piezoresistive sensors, with their low-cost-fabrication and easy data analysis advantages, have gathered significant attention.

Typically, sensors based on piezoresistivity rely on transducing external mechanical loading into resistance change, which usually follows a linear relationship [16]. Commonly, piezoresistive sensors harness the piezoresistive effect of the sensing material whereby its conductance changes with applied strain, along with change in resistance of the entire sensor assembly due to geometry change upon deformation. To design high-performance piezoresistive sensors, different parameters such as stretchability, sensitivity, dynamic range, limit of detection, accuracy, response speed, stability, durability, fabrication cost and simplicity should be considered. Out of these design criteria, the fundamental figure-of-merit for a strain sensor is its sensitivity, which is evaluated by the gauge factor (GF), formally defined as the ratio of relative resistance change in the sensing element to the mechanical strain acting on it (GF = ΔR/R/ε).

To date, various materials have been investigated for use as strain sensing elements, in an effort to optimize the response of the strain sensor with respect to the attributes mentioned above. Realizing a strain sensor operating at a wide strain range with good sensitivity has been an especially huge challenge. To overcome the problem of low sensitivity, different approaches have been proposed including doping, defect deformation and exploiting different piezoresistive sensing mechanisms along with sensing materials [17,18,19,20,21].

Among the most typical strain sensing materials are metals. However, strain sensors based on metals, otherwise known as metal-foil gauges, primarily rely on resistance change due to dimensional change of electrically conducting thin lines typically structured in the form of a serpentine, and as such the gauge factors are typically limited to single digits [14,22]. Several other strain sensors with different types of semiconductor piezoresistive materials, including doped polysilicon have also been developed, which offer much higher gauge factors compared to metal-foil counterparts [23].

As an alternative to some of these conventional materials like metals, metal oxides, semiconductors and ceramics which suffer either from intrinsic hardness, brittleness, low strain range or poor scalability, in recent years, carbon-based materials have been on the forefront of “sensor research”, including strain sensing [21,22,23,24,25]. As such, nanomaterials including carbon nanotubes (CNT) and graphene have both been reported as functional materials to realize strain sensors [24]. While CNTs have an almost one-dimensional (1D) structure [25], graphene has an ideal two-dimensional (2D) structure which potentially allows conventional device fabrication by planar, semiconductor process technologies. Additionally, its piezoresistive property [26,27] together with its exceptional physical, electrical [28] and mechanical properties (Young’s modulus on the order of 1 TPa) [29] render graphene an ideal candidate for strain sensors [30].

To date, there have been multiple studies which have reported graphene-based strain sensors built on a variety of substrates; however, the fabricated strain sensors display a wide range of gauge factors [31,32,33,34,35,36], making it inconclusive to verify the eventual advantages of graphene over standard metal-foil gauges suffering from low gauge factors. We argue that such variation in performance of graphene-based strain sensors is largely due to the problems in obtaining high-quality graphene, in a repeatable, uniform, scalable fashion and one that allows semiconductor process integration. Therefore, the intention of the present review on graphene-based strain sensors is to provide a critical perspective and discussion on the existing problems preventing the transition of “graphene strain sensors” into actual commercialization which are very much tied to: (a) the technology with which graphene is obtained, and (b) the integration of graphene into a realistic device topology.

Along these lines, in this work we critically survey the current state-of-the-art in graphene as a strain sensing material along with actual sensor demonstrations and relevant applications. To begin, we first discuss the fundamentals on graphene including electrical and mechanical properties, and the piezoresistive effect in graphene [26,27]. Then, we summarize the most common synthesis techniques of graphene and the three principal sensing mechanisms, to the extent that they are relevant, albeit not intending to be as comprehensive as review articles focusing specifically on these topics [37,38,39,40,41]. Next, detailed review and systematic discussion on graphene-based strain sensors is presented, along with a tabular summary of the different gauge factors (GF) and strain values achieved by the reported studies. We provide examples of real-life applications of graphene-based strain gauges along with recommendations and future outlook on the development of graphene-based strain sensors as well as the challenges that lie ahead.

## 2. Fundamental Material Properties and Piezoresistive Effect in Graphene

Graphene is a crystalline allotrope of carbon, which consists of a single-layer sheet of sp^2^ hybridized carbon atoms. After its exploration in 2004, graphene it has drawn a lot of attention due to its excellent electrical, mechanical, optical and magnetic properties [42,43,44,45,46,47]. In this section, we briefly discuss the fundamental material properties and major synthesis methods of graphene with the perspective to reflect on its potential as a piezoresistive material in strain sensing applications.

### 2.1. Electrical Properties

Studies on the electronic properties of graphene show that it is a new class of material resembling a zero-bandgap semiconductor and even acting more like a metal, yet still harboring the potential to have a bandgap and Fermi level by various methods, including doping [48]. Electronic properties of graphene also strongly depend on crystallite thickness. In single layer graphene, the band gap is zero, making it behave like a semiconductor or semi-metal, while the multilayer graphene shows metallic behavior as a result of the overlap in carrier wave function, which is due to the multiple graphene layers stacking [49]. The unique band structure of monolayer graphene leads to excellent traits, such as ballistic transport properties and anomalous quantum Hall effects, ultrahigh mobility (200,000 cm^2^/V·s) and high specific electrical conductivity (SEC) (0.95–1.67 S m^2^/g) [50], which can vary with applied strain.

Earlier study has shown the effect of the applied strain on opening the band gap of single-crystal graphene at the Fermi level, which results in the decrease of its electrical conductivity [51]. At low energies, graphene contains two linear energy bands that meet at high symmetric points and are isotropic with regard to the points at equilibrium. Effect of different strain types on the electronic properties of graphene reveals that, when isotropic strain is applied, graphene shows electronic properties that are independent of the isotropic strain since the isotropic strain follows crystal symmetries [52]. Strain can be intentionally or naturally imposed on graphene. By bending the substrates on which graphene is extended without slippage, uniaxial strain can be generated. To understand the effect of the uniaxial strain, armchair and zigzag graphene nanoribbons were studied, and they were reported to have different electronic properties. The electronic properties of the zigzag nanoribbons were independent of the uniaxial strain whereas the armchair nanoribbons were predicted to have energy gaps varying with the armchair shape [53].

### 2.2. Mechanical Properties

Graphene, as a two-dimensional one atomic layer thick material, sustains up to 25% in-plane tensile strain, making it one of the most flexible, uniform, zero band-gap semiconductors [54]. Graphene is known for its very high in-plane stiffness (high Young’s modulus), and the highest ever measured mechanical strength [44,55,56]. The 2D breaking strength and elastic stiffness of free-standing monolayer graphene membranes measured by an atomic force microscope (AFM) showed 42 N·m^−1^ and 340 N·m^−1^, respectively [44]; rendering graphene as the strongest material ever measured. These correspond to near theoretical limits including a mechanical stiffness of 1 TPa and an intrinsic tensile strength of 130 GPa at 25% strain, which are also comparable to in-plane values of graphite and single-walled and multi-walled carbon nanotubes [44]. It is important to note, however, that such mechanical properties largely depend on the testing temperature, sample geometry and even the measurement technique utilized. For instance, a layer of suspended exfoliated graphene located on a trench pattern of silicon oxide/silicon substrate was analyzed with AFM, showing graphene thickness of less than 10 nm, spring constant in the range of 1 to 5 N/m, and Young’s modulus of 0.5 TPa, which is less than that of bulk graphite typically ranging around 1 TPa [57]. Overall, the remarkable mechanical properties of graphene are very important, especially for flexible, stretchable electronics and/or wearable applications where robust and functional materials with excellent electronic and structural properties are needed [58].

### 2.3. Piezoresistivity

A piezoresistive effect is observed when a change in electrical resistivity of a material occurs as a result of applied stress. In other words, piezoresistivity is the change in resistivity of a material as a function of deformation. Germanium [59], silicon [60] and polycrystalline silicon [61] are the most common semiconductor materials that show a piezoresistive effect, and they are frequently used in MEMS for measurement of strain, pressure, acceleration, flow and tactile sensing, as well as haptics applications.

Graphene has attracted a lot of attention, not only due to being the thinnest known material and having unique electrical and mechanical properties, but also due to having a linear change of resistance versus strain, making it a good candidate for piezoresistive sensor applications [62]. In this regard, piezoresistivity of multilayer graphene on poly (methyl methacrylate) (PMMA) substrate was investigated through a bending test that showing high piezoresistivity with a gauge factor of 50, demonstrating the potential of graphene for strain sensing applications [63]. In addition, Anderson D. Smith et al. verified the piezoresistive effect in graphene by applying uniaxial and biaxial strains [64]. Gauge factors of biaxial strained devices were found to be higher than that of uniaxial ones.

The piezoresistivite effect in graphene has been elucidated with three different mechanisms which include: (a) structure deformation, (b) over-connection of graphene sheets, and (c) the tunneling effect among neighboring sheets.

(a) Structure Deformation

**Figure 1:**
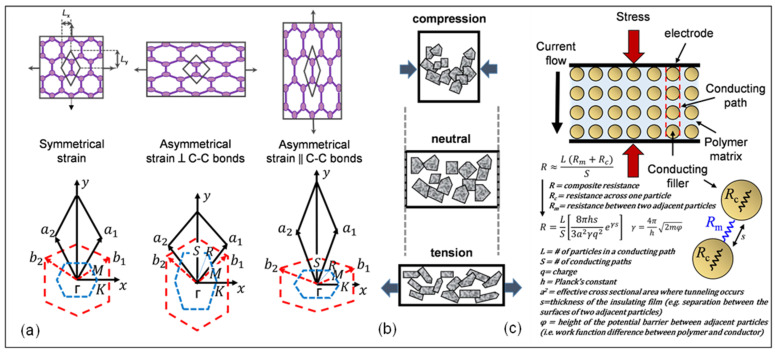
Piezoresistivity mechanisms of graphene: (**a**) symmetrical strain distribution, asymmetrical strain distribution perpendicular to C-C bonds and asymmetrical strain distribution parallel to C-C bonds [51]; (**b**) schematic illustration of piezoresistivity of graphene sheets [71]; (**c**) schematic illustration of the tunneling model. Reprinted with permission from ref. [72]. Copyright Clearance Center.

Electrical-mechanical coupling in graphene can be observed when significant elongation in graphene causes changes in its electrical properties and band structure. Recent studies on strained graphene demonstrate that changes in electrical properties of graphene are related to the type of strain distribution. In symmetrical strain distribution, additional scattering and resistance decrease is observed while no change occurs in other graphene properties such as band-gap opening [65,66,67,68,69,70]. On the other hand, asymmetrical strain distribution in graphene results in opening of band gaps at the Fermi level, which is explained by pseudo-magnetic field. Strain distributions in graphene significantly modify the band structure of graphene around the Fermi level, resulting in remarkable change of the pseudogap width in the case of symmetrical strain distributions and band-gap opening in the case of asymmetrical strain distributions. The band gap is enlarged by increasing the amount of strain, reaching a maximum value of 0.486 eV at 12.2% strain parallel to C-C bonding, and to a maximum of 0.170 eV at 7.3% strain perpendicular to C-C bonding (Figure 1a) [51].

(b) Over-connected Graphene Sheets

As shown in Figure 1b, a larger sheet of graphene can be thought of as a conductive network of smaller connected sheets or flakes. From a nanoscopic perspective, the distortion of a small graphene sheet alters the resistivity of the single sheet, which can consecutively trigger a resistance change in the entire conducting system. Thus, the stress response of the graphene network relies primarily on the contact strength of the neighboring plates from a macroscopic point-of-view. Overlap area and contact resistance determine the conductivity between the neighboring flakes. As displayed in Figure 1b, the overlap between neighboring flakes becomes smaller or greater such that the resistance changes upon tensile or compressive loading making graphene a suitable material for strain sensing applications [30,71].

(c) Tunneling Effect among Neighboring Graphene Sheets

It is known that the distance between two graphene sheets specify the conductivity of graphene. Due to the tunneling effect, current can flow from one single graphene sheet to another. As a result, the resistance increases exponentially and proportionally with the distance (Figure 1c) [72]. This mechanism can be used to achieve higher GF in graphene-based strain sensors. As shown in Figure 1c, by assuming that the resistance of the matrix is constant everywhere, the resistance of the paths perpendicular to the current flow can be ignored, and thus the number of conducting particles between electrodes, as well as the number of conducting paths, becomes a factor in this relationship. The total resistance can then be calculated as R, which is shown in Figure 1c.

## 3. Methods of Obtaining and Transferring Graphene

Despite its superior electrical and mechanical properties, the challenges in obtaining pristine graphene limit the widespread use of this 2D material in device applications. In an effort to address this problem, numerous techniques were investigated to obtain thin graphitic films and few layer graphene (FLG). Initial demonstrations primarily through mechanical exfoliation followed by transfer of graphene onto silicon substrates, marked a major breakthrough in graphene research [73]. Even though mechanical exfoliation (i.e., Scotch tape method) provides the highest quality graphene, this approach has some disadvantages such as depending largely on the hand skills of the researcher, lack of repeatability and scalability, as well as limitations on graphene flake size and shape being small and irregular.

**Figure 2:**
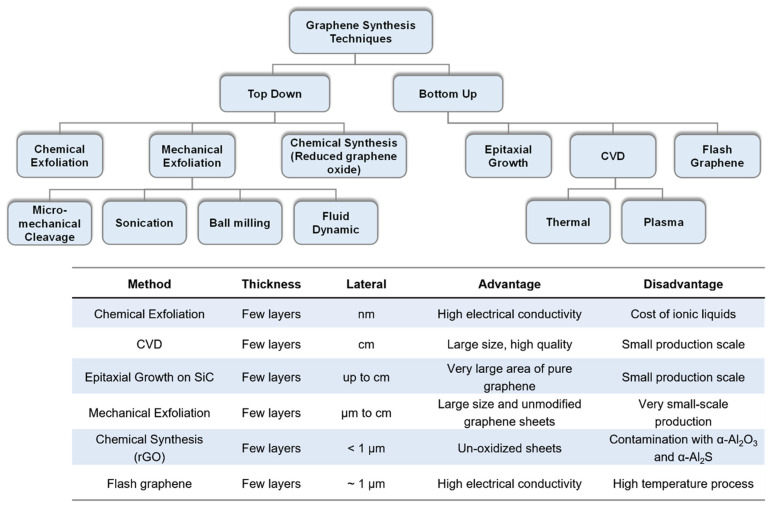
Overview of the most common techniques to obtain graphene categorized based on top-down and bottom-up processes, along with a tabular comparison on the thickness, lateral size, fundamental advantages and disadvantages of each technique. Reprinted with permission from ref. [74].

Therefore, research on obtaining high-quality graphene along with its integration to different substrates which often requires transfer methods, has received serious effort especially over the past two decades. The method with which graphene is obtained directly affects the quality of graphene including its electrical, mechanical and piezoresistive properties. Different methods which are classified as bottom-up and top-down processes have been utilized in order to obtain high-quality graphene. The most commonly used methods are: chemical exfoliation [74], chemical vapor deposition (CVD) [75], epitaxial growth [76], mechanical and reduction of graphene oxide rGO [77,78], and flash graphene synthesis [79]. Graphical overview of these techniques along with the major advantages and drawbacks of each approach are summarized in Figure 2.

### 3.1. Chemical Vapor Deposition (CVD)

This approach, which is classified as a bottom-up method, can be used to produce mono- or multiple-layers of graphene on a catalytic transition metal substrate. Since its first demonstration in 2006, CVD-based synthesis of few-layer graphene (FLG) witnessed continuous progress transitioning into an established method today for providing scalable and reliable production of high quality, large-area graphene [75,80,81,82]. In CVD method, different catalytic transition metals such as copper (Cu) [75,83], nickel (Ni) [84,85], ruthenium (Ru) [86], or cobalt (Co) [87,88] are used as catalysts to grow mono- or multiple layers of graphene. Among the various metal catalysts, copper is the most widely used material for graphene synthesis, as it promises low-cost production on flexible Cu foils which can be lined up in the interior of the growth chamber typically a quartz tube. Besides, carbon solubility is particularly low (0.03 atom%) at the standard graphene growth temperatures (1000–1060 °C) [89].

**Figure 3:**
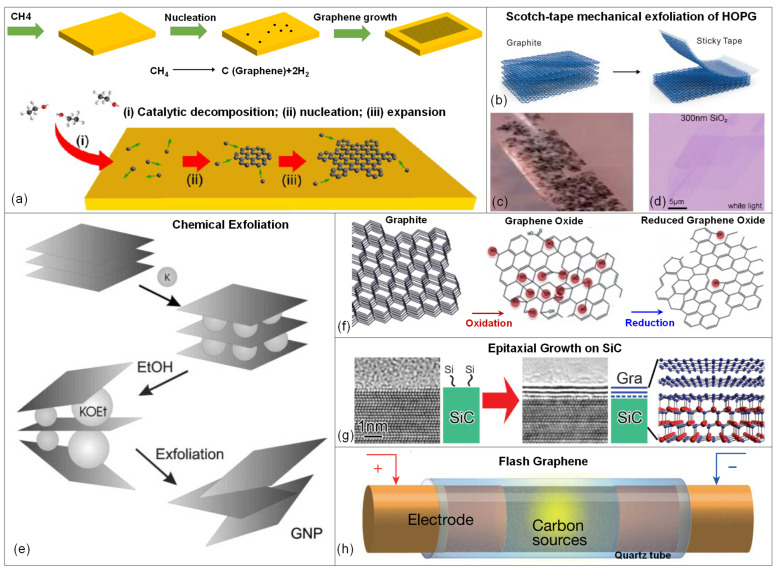
Methods for synthesizing graphene (**a**) Schematic of the initial state growth of graphene on copper from ethanol-CVD method. Adapted with permission from ref. [90]. Copyright (2013) American Chemical Society. (**b**) Schematic illustration of the mechanical exfoliation [92]. (**c**) An image of graphene flakes on scotch tape [93]. (**d**) Optical microscopy image of relatively large few layers of transferred graphene flakes on a SiO_2_/Si. Reprinted from ref. [94], with the permission of AIP Publishing. (**e**) Schematic diagram of chemical exfoliation by ethanol to form graphene nano plates [95]. (**f**) The schematic diagram of reducing graphene oxide to develop reduced graphene oxide [96]. (**g**) Epitaxial growth of graphene on a SiC wafer [97]. (**h**) Schematic of flash Joule heating (FJH) [79].

During synthesis, detachment of carbon atoms from methane gas (CH_4_) source takes place on the surface of Cu substrate to form the graphene lattice. Graphene islands, which show different lattice orientations in atomic scales enlarge and grow together on the substrate (Figure 3a) [90,91]. Various factors influence the graphene growth on a catalyst, such as system pressure and temperature, crystal structure, lattice parameter and carbon solubility in the metal.

CVD technique provides many opportunities such as low cost, easy film transferring, employment of various transition metals as catalysts, and the ability to produce large-area films with high uniformity and low defects, which makes it a promising route to obtain graphene [98]. Despite the advantages, graphene obtained through chemical vapor deposition (will be referred to as “CVD graphene” hereafter) has less mobility, higher impurity doping and greater asymmetry in electron and hole concentration. In addition, the requirement for a metal catalyst creates a bottleneck in direct use of this as-grown graphene layer. The removal of the catalyst layer can cause degradation in graphene film quality, as well as further problems due to use of etchants such as ferric chloride (FeCl_3_) and complexities during transfer of graphene to receiving substrates (e.g., silicon). All in all, this makes fabrication of sensors (including strain gauges) and electronic devices in general, difficult on CVD graphene.

### 3.2. Mechanical Exfoliation

One of the top-down techniques for synthesizing graphene is exfoliation. Mechanical and chemical methods are the two types of this technique. The first graphene was obtained by mechanical exfoliation or tape-peeling method, also known as the Scotch tape method, from a highly ordered pyrolytic graphite (HOPG) in 2004 by Novoselov and Geim (Figure 3b–d) [99]. Although mechanical exfoliation of graphene with tape is not feasible for large-scale production, pristine graphene produced by this method is of high quality, has high mobility of ~10,000 cm^2^/V·s at room temperature and the approach is low-cost with minimum investment on experimental setups [63]. Additionally, graphene is directly obtained from HOPG, meaning that there is no requirement on the use of metal catalysts neither their subsequent removal with chemical etchants like iron nitrate (Fe(NO_3_)_3_) [100], iron chloride (FeCl_3_) [101] and ammonium persulfate ((NH_4_)_2_S_2_O_8_) [80], which are harsh, environmentally hazardous and/or expensive to dispose of.

In order to optimize the mechanical exfoliation method for obtaining high-quality graphene, different studies have been performed to investigate the underlying mechanisms in exfoliation [102]. Mainly, there are two means to mechanically exfoliate graphite into graphene flakes. The first one uses normal or shear force to overcome the van der Waals bonds between the graphene layers in the bulk graphite. The other way is the fragmentation of large graphite layers to smaller ones, after which it becomes easier to exfoliate the smaller graphite flakes and gradually obtain a layer of graphene. However, this method is not suitable for achieving large-area graphene.

Different types of mechanical exfoliation techniques such as micromechanical cleavage [99], sonication [77], ball milling [103,104,105,106,107,108] and fluid dynamics-assisted exfoliation [109] have used these mechanisms. Recently, a new technology by Lynch-Branzoi et al. was introduced to produce graphene enhanced polymer matrix composites (G-PMCs) which have been used for in-situ shear exfoliation of mined graphite directly within molten thermoplastic polymer. In this study, the raw material is graphite, and functionalization arises between polymer and graphene nanoflakes [110].

### 3.3. Chemical Exfoliation

Chemical exfoliation is a technique that exfoliates solution dispersed graphite by injecting large alkali ions between the graphite layers (Figure 3e). This procedure involves the preparation of a solution that converts graphite to graphene by the synthesis of graphene-intercalated compounds [111]. Chemical exfoliation includes graphite exfoliation in a solution and typically consists of two phases. First, the space between layers of graphene is expanded by decreasing van der Waals forces. The final phase is to split or exfoliate graphene into mono- or few layer graphene using sonication or fast heating [74]. Chemical exfoliation is a critical and unique method for synthesizing graphene since it can create a high volume of graphene at a low temperature. Additionally, it is scalable and may be used to a broad variety of functionalized graphene manufacturing processes.

### 3.4. Reduced Graphene Oxide (rGO)

Chemical oxidation of graphite with the use of various oxidants to produce graphene oxide (GO), and the subsequent removal of oxygen functional groups from GO to obtain reduced graphene oxide (rGO), which is considered to be a form of graphene, is a widely used approach especially when large quantities are needed [96]. Typically, the modified Hummer’s method is used, where graphite flakes are suspended in a solution of potassium permanganate (KMnO_4_), sodium nitrate (NaNO_3_) and sulfuric acid (H_2_SO_4_) [112,113] to produce graphene oxide. GO can then be converted into rGO through a chemical, thermal, microwave or photo reduction technique [114,115,116,117].

Reduction of graphene oxide, illustrated in Figure 3f, ends up with some defects degrading the crystallinity and lowering the electrical conductivity of graphene compared to that of pristine graphene which could be obtained through mechanical exfoliation [117]. However, since rGO is obtained by a cheap and simple process, and with the increasing demand on low-cost and scalable devices, reduced graphene oxide still stands out as a good alternative for the fabrication of graphene-based devices, including strain sensors [118].

### 3.5. Epitaxial Growth

Epitaxial growth on silicon carbide (SiC), is another method to obtain relatively large areas of graphene via thermal decomposition of bulk SiC due to the difference in silicon and carbon’s vapor pressure [71,92,114,115]. By heating a single crystalline SiC wafer to a temperature above 2000 °C in vacuum or inert gas (such as argon) atmosphere, silicon atoms are decomposed from the (001) plane of the crystal surface and the remaining carbon atoms form the epitaxial graphene (EG) on the surface, spontaneously (Figure 3g). The number of graphene layers can be controlled by manipulating the process parameters. Since SiC is commercially available, this renders epitaxial growth of graphene a suitable technique for device applications [119,120,121,122,123,124]. Another advantage of this technique is that the high quality and homogeneous graphene has exceptionally high electron mobilities, which makes it desirable in high-speed electronic devices. Recently, different substrates, such as ruthenium [125,126,127], iridium [128], copper [75,129], platinum [130] and Ni thin film [131] were also reported to attain high quality EG.

### 3.6. Flash Graphene

Flash graphene is a green technique that manufactures pure graphene in large quantities from waste food, plastic, and other components. This technology has the capability of converting almost any carbon source into graphene flakes. The procedure is rapid and inexpensive; using the flash graphene technology, a ton of coal, food waste, or plastic can be converted into graphene for a fraction of the cost which is used by conventional graphene production techniques. By heating carbon-containing materials to 3000 Kelvin (about 5000 degrees Fahrenheit), flash graphene is created in 100 ms [132]. The high temperature is critical to the technique and typically it is three times those encountered in chemical vapor deposition approach. In this process, amorphous conductive carbon powder is softly squeezed into a quartz or ceramic tube between two electrodes (Figure 3h). Copper, graphite, or any other conductive refractory material can be used as electrodes, and they should fit loosely inside the quartz tube to allow for outgassing during flash graphene process. In less than 100 ms, a high-voltage electric discharge from a capacitor bank heats the carbon source to temperatures in excess of 3000 K, successfully transforming the amorphous carbon to graphene [79].

### 3.7. Transfer and Integration of Graphene with Device Substrates

Especially for CVD graphene, a “transfer” step is employed where the metal catalyst on which graphene is grown is removed, and the released graphene layer is transferred onto a receiving substrate like silicon. However, the ultrathin CVD-grown graphene is too sensitive to rip and tear during etching and transfer, and depending on the quality of the synthesized graphene, even a very small disturbance could suffice to break the film apart. To address this issue, a widely used transfer method is “polymer-supported metal etching”, where a polymer layer is employed to mechanically support CVD-grown graphene, even HOPG [133] and graphene oxide (GO) [134] during subsequent process steps including wet etching of nickel or copper catalyst layers. Polymer-assisted transfer with materials like PMMA has become effective in facilitating the safe transfer of graphene, and in many studies it has become preferable over alternative strategies such as dry transfer [80]. Using polymer-supported transfer, large area CVD graphene up to several inches in lateral width is possible, offering adequate room for post-processing of devices including strain gauges where an individual sensor could easily occupy a few millimeters in width and length.

**Figure 4:**
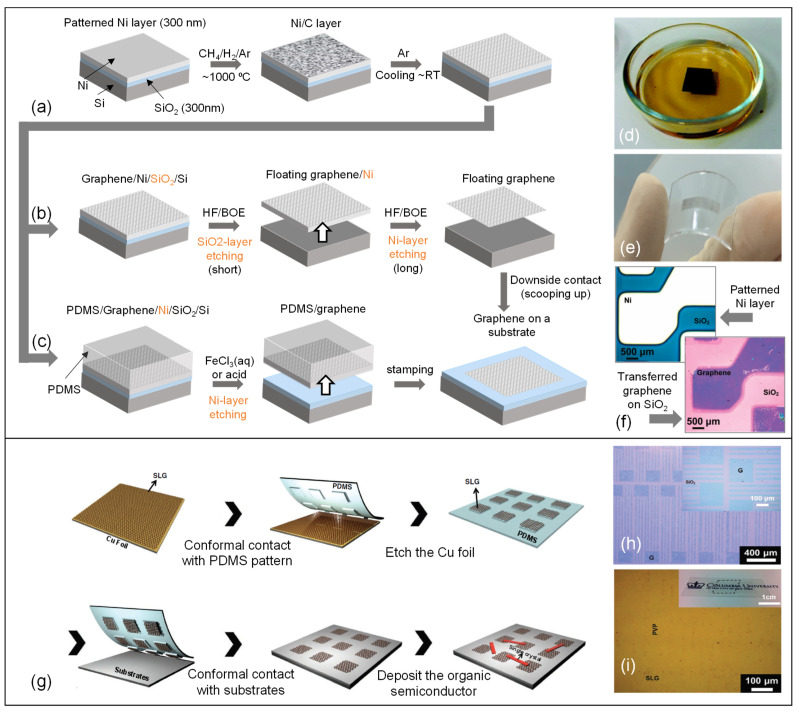
Dry transfer process for a graphene film grown on a Ni film using a soft substrate, PDMS. (**a**–**c**) Schematic illustration of synthesis, etching and transferring processes of graphene films without and with a PDMS stamp. Reprinted with permission from ref. [101]. Copyright (2009) Springer Nature. (**d**) Using FeCl_3_ solution to etch underlying Ni layer. Reprinted with permission from ref. [137]. Copyright (2009) Royal Society of Chemistry.(**e**) Transparent graphene films on the PDMS substrate. Reprinted with permission from ref. [138]. Copyright (2017) Elsevier (**f**) Optical microscope image of a patterned nickel layer on which graphene is grown and image of the graphene layer on a SiO_2_ substrate following successful transfer. Adapted with permission from ref. [133]. Copyright (2009) American Chemical Society. (**g**) A schematic of micropatterned single layer graphene transferring process to a substrate. (**h**) An optical microscope image of patterned SLG electrode on SiO_2_. (**i**) An optical microscope image of patterned SLG electrode on PET/graphene/PVP. Reprinted with permission from ref. [135]. Copyright (2011) John Wiley and Sons.

Typically, ferric nitrate (Fe(NO_3_)_3_), ferric chloride (FeCl_3_), ammonium persulfate ((NH_4_)_2_S_2_O_8_) are used to etch Ni and Cu metal layers away from the surface, without needing a polymer support. Transferring a layer of CVD graphene (Figure 4a) to a Si/SiO_2_ substrate was reported in which wet etching of SiO_2_ and Ni layers was performed by BOE and FeCl_3_ solutions, respectively (Figure 4b) [101].

Another preferable material for transferring graphene to a substrate is polydimethylsiloxane (PDMS). Promising properties of PDMS such as durability, nonreactivity, moldability, solvent resistance, and most significantly the low surface free energy make it an excellent candidate for soft lithography [88,132,133]. The low adhesion force between the PDMS and the applied substance on the PDMS, helps the substance to be released from PDMS when it is stamped onto a target substrate. In addition, PDMS protects graphene from mechanical defects during the transfer process until the metal substrate etching is complete (Figure 4c) [101]. SiO_2_/Si and polyethylene terephthalate (PET) are known as typical substrates in soft lithography to receive graphene from PDMS. Figure 4d–f display samples of both etching and PDMS transfer processes and transferred CVD graphene film on a SiO_2_ substrate.

PDMS is also useful for fabricating graphene-based devices by stamping method [93]. Growing a patterned graphene by using a pre-patterned metal catalyst layer should be done very carefully, otherwise ruptures may occur on the surface of graphene which changes electrical and mechanical properties of the final device substantially. On the other hand, PDMS stamping technique not only eliminates the performance degradation in the graphene layer, but also facilitates the fabrication of graphene-based devices. Kang et al. reported a successful device fabrication using PDMS stamp, which is shown schematically in Figure 4g. The molded PDMS with desired pattern have been used to grow patterned graphene [135]. The patterned PDMS was stamped onto the metal/graphene surface, then by etching metal layer, only the graphene layer was left on the patterned PDMS, which is feasible to be stamped onto other substrates to construct transparent conductive electrodes or enable the fabrication of organic field-effect transistors (Figure 4h,i). Yet another example capitalizing upon transfer approaches is a strain sensor for electronic skin applications wherein quasi-continuous nano graphene film was merged with flexible substrates resulting in a very high sensitivity, long lifetime and fast response [136].

## 4. Graphene-Based Strain Sensors

There are a number of studies in which graphene is used as a strain sensing material. Typically used to fabricate flexible graphene-based strain sensors, graphene can be compounded with elastomers to realize flexible strain sensors with sufficient piezoresistive performance, owing to the excellent electro-mechanical properties of graphene, along with the stretchability and flexibility of polymer matrix. A number of polymers have been utilized in strain sensor applications, where flexibility and stretchability factors are concerned in order to obtain high sensitivity with robust mechanical strength. In this regard, PDMS, PET, 3M elastic adhesive tape, PU and natural rubber have been employed to fabricate graphene-based strain sensors [139].

The performance, more specifically the gauge factor, of these polymer integrated graphene-based strain sensors varies due to different forms of graphene (as detailed in Section 3) and their implementation methods in polymeric/elastomer matrix structures. We therefore classify graphene strain gauges based on the three most common methods with which graphene is obtained namely: (a) CVD, (b) exfoliation, and (c) reduction of GO. Tabular summary of existing strain gauges based on the three different forms of graphene is provided in Table 1, along with their gauge factor and strain range as performance metrics. In the discussion to follow, we elaborate on graphene strain gauges based on this classification methodology.

### 4.1. CVD Graphene-Based Strain Sensors

Strain sensors based on CVD-grown graphene integrated into various substrates including flexible films or polymers (including graphene-loaded polymers and graphene nanocomposites) as well as suspended graphene structures, either with or without a backing membrane, are widely reported in the literature, offering a wide range of gauge factors ranging anywhere from single digits up to a million.

**Figure 5:**
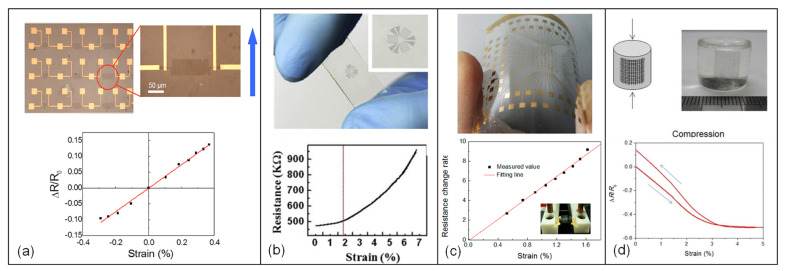
CVD graphene-based strain gauges: (**a**) optical microscope images of the as-patterned devices with the zoom-in image of an individual device (arrow marks the bending direction) along with a plot of the resistance change normalized to baseline resistance (ΔR/R_0_) versus applied strain. Reprinted with permission from ref. [140]. Copyright (2012) AIP Publishing. (**b**) photograph of transparent graphene strain sensor and variation of resistance with respect to stretching up to 7.1% for graphene strain sensor. Reprinted with permission from ref. [143]. Copyright (2013) Elsevier. (**c**) photo of “e-skin” with an 8 × 8 device array and resistance change rate with an increase in applied strain. Adapted with permission from ref. [136]. Copyright (2015) American Chemical Society. (**d**) compression of universal strain sensing and corresponding changes in resistance under different deformation. Reprinted with permission from ref. [144]. Copyright (2012) Elsevier.

Among the various studies, monolayer CVD-grown films have been demonstrated to exhibit high gauge factors. For example, nanographene (NG) sheets with high gauge factors for ultra-sensitive strain sensors have been reported [140]. The suggested NG-based strain sensors used charge tunneling between nearby NG islands, thereby having significant piezoresistive sensitivity under a tensile or compressive strain. A very sensitive conductor network is used in the NG film, resulting in an extremely high gauge factor of more than 300 (Figure 5b). NG films with remarkable sensitivity and low resistance were also transferred onto flexible substrates for force mapping applications. A high gauge factor of more than 500, a long lifetime of more than 104 cycles, and a rapid reaction time of less than 4 ms were achieved [136] (Figure 5c).

In another study, graphene woven fabrics (GWFs) were explored as a potential candidate to achieve very high sensitivities. GWFs were created by growing graphene on the surface of crisscross copper meshes using atmospheric pressure CVD technique. Results showed that the GWFs can have incredibly high gauge factors, reaching ~10^3^ for 2–6% strains and 10^6^ for larger strains (>7%), and 35 for very small strains of 0.2%. Due to its woven mesh design and fracture behavior, the electrical resistance of GWFs grows exponentially with tensile strain. To reveal the potential of GWFs for use in tensile strain sensors, strain sensing experiments using GWF-on-PDMS sensors, such as compression was performed, which is shown in Figure 5d along with corresponding changes in resistance under different deformations. Result show the highest so far GF recorded, rendering this design a promising choice for sensing tensile deformation by changes in strain [144].

There are some other methods to obtain high gauge factor as well as high durability of the sensor. For instance, braided graphene belts (BGBs) based strain sensor have been prepared which possess a wide sensing range of up to ~55% tensile strain and a reliable and linear resistance change up to 35% strain, which gives a high GF of 175.16. It also shows a high cyclic repeatability (>6000 cycles) at 10% and 30% strain. The high sensitivity and wide range of this strain sensor is supposed to be related to the orientation and intersection of BGB sensing belts that are the regions of stress concentration where the crack can rapidly grow [149]. Similarly, another strain sensor that can bear large strains and also achieve a high GF was constructed by connection of highly sensitive planar graphene and highly stretchable crumpled graphene (CG) films [150]. The CG films were achieved by transferring CVD graphene to a pre-stretched very-high-bond (VHB) substrate. The device was tested in bending and stretching modes with resulting GF of 20.1 with 105% tensile strain, and GF of 337.8 in strain range of 105–135%.

Graphene based devices that can be conformably bonded to a highly deformed surface and maintain stable electrical and mechanical properties during severe external deformation can be used in wearable electronic applications including fitness trackers, smart medical gadgets, and health monitoring systems [147]. In an effort to create a highly flexible and sensitive strain sensor, a combination of fragmented graphene foam (FGF) and polydimethylsiloxane (PDMS) is utilized. The stated strain sensor has a high sensitivity with a gauge factor of 15 to 29, which is significantly greater than the GF/PDMS strain sensor’s gauge factor of 2.2. Aside from its great sensitivity, the FGF/PDMS strain sensor has a high stretchability of over 70% and a high durability of over 10,000 stretching-releasing cycles [146]. Yet another demonstration enabling a wearable application such as a musical instrument, is a strain sensor based on GWF/PDMS composite offering both exceptionally high GFs (223 at a strain of 3%) and great flexibility, and the capacity to detect multi-mode deformations such as tensile and flexural stresses [145].

Alternative to integration on flexible substrates, graphene can also be integrated on membranes formed atop rigid semiconductor substrates. For instance, CVD-grown multilayer, polycrystalline graphene was transferred onto a silicon nitride membrane to create graphene-based piezoresistive pressure sensors. Strain on the graphene layer was obtained by exerting differential pressure across the membrane. According to the results of electromechanical experiments, graphene subjected to a tensile strain of ~0.25% displayed a gauge factor of ~1.6 [23]. To enhance strain in the graphene membrane, a novel sensor design based on an array of holes etched into a supporting nitride membrane was utilized, where strain was raised by local deformations of the holes under an imposed differential pressure [142]. The graphene membrane achieved a gauge factor of 4.4, at a sensitivity of 2.8 × 10^−5^ mbar^−1^ with high linearity throughout the whole pressure range. For a 14.3 µm deflection at the membrane’s center, the average strain of the suspended square membrane was determined to be 0.22%.

While maximizing the GF and strain range are critical considerations, another important criterion in sensor response is the linearity between the gauge factor and the applied strain. As an example, Bae et al. [143] investigated a transparent and stretchable strain sensor, discovering two distinct regions for the sensor operation: (i) a virtually linear connection between resistance change and strain when the applied strain is less than 1.8%, and (ii) a nonlinear relationship when the strain is between 1.8% and 7.1% (Figure 5a). Accordingly, the gauge factor was around 2.4 in the first region, which is close to that of a traditional metallic strain gauge, whereas in the second region for strain values in excess of 1.8%, GF varied from 4 to 14. Additionally, in another study, a transparent strain sensor was fabricated based on a hybrid material of graphene and g-C_3_N_4_ heterostructure on PDMS substrate, where the calculation shows the sensor has linear response to tensile and compressive strain by suggesting band-gap opening from 0.19 eV to 2.46 eV in a wide range of strain (−12% to 20%) with a GF of 1.89 [151].

Another important issue for graphene-based strain sensors is their scalability and applicability for large scale manufacturing. To potentially address this problem, an approach for synthesizing and transferring highly conductive and transparent wafer-scale graphene sheets was presented by Lee et al. [148], and strain gauges achieving GF of ~6.1 (at applied strain up to 1%) were demonstrated. This is a relatively big gauge factor for large-scale transferred graphene which at the same time outperforms typical GF values of metal foil gauges, and justifies the potential feasibility of CVD-grown graphene for strain gauge implementation provided that integration challenges are fully addressed.

### 4.2. Exfoliated Graphene-Based Strain Sensors

**Figure 6:**
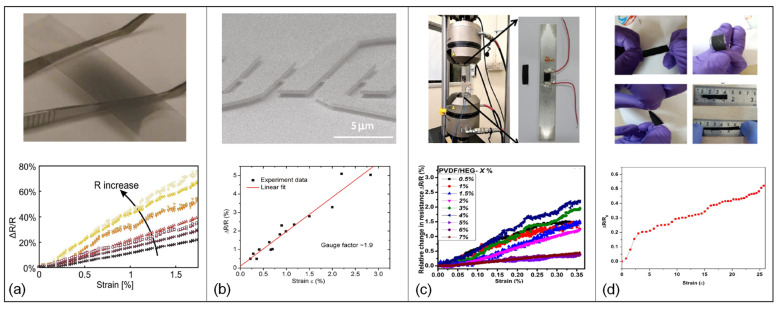
Exfoliated graphene-based strain gauges (**a**) Sample of graphene film and curves of normalized change in electrical resistance versus strain for several strain gauges. Adapted with permission from ref. [154]. Copyright (2012) American Chemical Society. (**b**) SEM image of four suspended graphene devices made from a single flake and the electrical measurements of uniaxially strained graphene (relative change of resistance as function of strain). Adapted with permission from ref. [159]. Copyright (2011) American Chemical Society. (**c**) Piezoresistive measurement of the fabricated strain sensor on the aluminum specimen, which is under uniaxial tensile loading and relative change in resistance against applied strain [161]. (**d**) Optical photographs of fabricated GNP/PDMS strain sensor at stretchable, foldable, twistable and demonstration of fabricated GNP strain sensor and relative resistance versus strain [162].

Several attempts have been made to use exfoliation for fabricating extremely dependable and sensitive graphene-based thin film strain gauges. Hempel et al. [154], for example, described a novel strain gauge based on thin films of overlapping graphene flakes. A cost-effective and scalable fabrication method which at the same time offers high gauge factors (150) that could be controlled by altering the film morphology caused by deposition was demonstrated. Image of an exfoliated graphene-based strain gauge formed on a PET substrate, and its corresponding normalized change in resistance versus strain for different strain gauge realizations depending on the deposition-controlled morphology of the graphene film is shown in Figure 6a.

To obtain high GF with small applied strains in exfoliated graphene-based strain sensors, Casiraghi et al. [158] used ultrasonic-assisted liquid phase exfoliation in water to create graphene ink from graphite, after which inkjet printing was used to create graphene strain gauges on paper. A maximum GF of 125 was achieved, which is linked to high sensitivity, even when small strains (0.3%) are used.

In another experiment, a flexible and hydrophobic sensor with excellent durability and high gauge factor (~1000) in strain range of 0.05% to 0.265% was realized by using 10 mL of graphene solution coated on polypropylene film by layer-to-layer method [160]. Also, another waterproof and flexible strain sensor was fabricated by utilizing poly(vinylidene difluoride) (PVDF) as the matrix with a polymer-functionalized hydrogen-exfoliated graphene (HEG) as nanofillers in the matrix [161]. In Figure 6c, the relative resistance change of the fabricated strain sensor mounted on an aluminum specimen subject to uniaxial tensile load is shown, wherein, by using a low concentration of nanofillers, a maximum gauge factor of 10 was achieved.

For detecting large levels of strain especially in some wearable applications, flexible substrates have been used along with graphene as the sensing material to implement stretchable strain sensors. For instance, graphene nanoplatelets (GNP) were used on different flexible substrates such as PDMS, PET, polyimide and carbon fiber to fabricate resistive-strain sensors in order to detect various human body motions [162,163,164]. Images of the fabricated GNP/PDMS strain sensor during stretching, folding and twisting conditions along with a plot of the relative resistance change versus strain are shown Figure 6d. The GNP/PDMS strain sensor demonstrated a fast response time and good sensitivity (GF of 62.5) that is linear in high ranges of stretching (2.5% to 25%), with excellent repeatability and stability. Likewise, a new strategy was recently employed to realize a pressure sensor by using graphene foam (GF)-PDMS nanocomposite that shows ultrasensitive piezoresistive behavior [166] where gauge factor up to 178 was achieved at 10% compressive strain.

A critical aspect in exfoliated graphene-based strain sensors is the actual number of graphene layers which directly impact the gauge factor. The gauge factors of strain gauges comprised of different graphene layer counts were evaluated using the equivalent stress beam. to investigate the influence of single- and multi-layer graphene sheets. When a concentrated force was applied on the end of the cantilever and the strain was varied from 0% to 0.084%, the gauge factor ranged from 10 to 15, depending on the number of layers in the graphene sheet [155]. Another study examined the effect of graphene with a higher layer count up to six, and among these tri-layer graphene showed the most pronounced response. The gauge factor was calculated to be in the range of 0.6. In this study, suspended graphene membranes were created by employing mechanical exfoliation process followed by integration of graphene to pre-defined trenches etched into 300-nm thick SiO_2_ wafers [156].

Another point of consideration is the variation of gauge factor with respect to different types of applied strain. When uniaxial tensile strain is applied to suspended graphene devices, electrical tests show that the gauge factor of graphene is 1.9. A moderate uniaxial strain was shown to be incapable of creating a band gap in graphene and has no effect on its carrier mobility. SEM imagery of four suspended graphene devices made from a single flake and the relative change of resistance as a function of strain is presented in Figure 6b. In this sort of experiment, the highest achieved tensile stresses are predicted to be ~2–3%, well within the elastic-only regime [159]. In case of higher applied strain through vertical deflection with an AFM tip, Benameur et al. [157] detected oscillations in the electromechanical response of bilayer graphene. As such, the upper limit of the gauge factor was determined by accounting for the uniform strain caused by the vertical deflection while ignoring the contribution concentrated near the AFM tip, and an upper limit for GF of 8.8 was found at strains up to 5%.

Effects of structural deformations in the graphene layer versus its strain sensing properties have also been investigated. Accordingly, the change in resistance of both a rippled graphene device and a buckled nanographene film device were compared under different tensile strains [165]. The rippled graphene has shown a negative trend in resistance change when the amount of applied strain was gradually increased from 0% to 20%, which was attributed to the geometry of graphene, such that the higher the strain, the higher the conduction paths exist. GF of −2 was obtained for this device. Contrary to that of rippled graphene, a buckled nanographene film has shown an increase in its sheet resistance when experiencing a strain of 0% to 30%, which is due to re-arrangement of nanographene domains as they overlap and further compact after buckling. Accordingly, when subjected to tensile strain, resistance of rippled graphene yields a positive gauge factor of 0.55.

Analyzing the literature, we see that through potential advancements in exfoliation approaches that could enable accurate layer control with minimal defects, exfoliated graphene bringing in the advantage of pristine material quality can be a viable route to realize strain gauges with high gauge factors.

### 4.3. rGO-Based Strain Sensors

Oxidation of graphite to create graphene oxide followed by a chemical reduction step provides graphene flakes, also referred to reduced graphene oxide (rGO), typically suspended in a solution which could be water or a suitable solvent. Unlike CVD graphene or exfoliated graphene, since rGO is in “solution phase”, building a realistic device out of rGO calls for different fabrication and integration approaches, including strategies used in flexible electronics manufacturing. For instance, PET substrates were drop-casted with GO and reduced using the laser source of a commercial laser printing machine where the optimal laser power for reduction was determined as 1.8 W. The resulting strain gauges displayed a gauge factor of 61.5, wherein the recorded resistance values for applied strains ranging from 0.01 to 0.04% were relatively linear for each measurement sequence [167].

Considering an rGO-based graphene film as a network of over-connected rGO fragments where the applied strain causes separation in the junctions and causes resistance change, one could envision that by controlling the rGO film thickness and tailoring the physical separation between rGO fragments, excellent sensitivity to mechanical stimuli can be achieved. Based on this fundamental principle, various strategies have been followed to realize highly sensitive strain sensors which at the same time could be flexible and/or stretchable by way of selecting suitable substrate materials.

One such approach relied on doping the rGO film with polystyrene nanoparticles, which significantly change the physical stacking of rGO fragments and therefore upon deformation a much more pronounced resistance change is observed. Accordingly, even under small strains of 1.05% GF values of 250 could be obtained [174]. Likewise, a low-cost flexible strain sensor was fabricated to monitor micro strain level structural variations by using rGO mesh film on a liquid crystal polymer (LCP) substrate, where the developed sensor achieved high sensitivity (GF 375–473), good stability and high reversibility based on a mechanism of high-density crack formation under tensile strain [176].

Similarly, a two-part, crack-based strain sensor employing silver nanowires/graphene hybrid particles which exhibited gauge factors as high as 20 for strain changes (Δε) less than 0.3%, 1000 in the 0.3% < Δε < 0.5% range, and 4000 in the 0.8% < Δε < 1% strain range. This strain gauge also displayed high sensitivity to bending, high strain resolution and high operating stability, and it has been effectively utilized in the detection of micro strains including daily physical vibrations, wrist pulses and recognition of sound [171].

**Figure 7:**
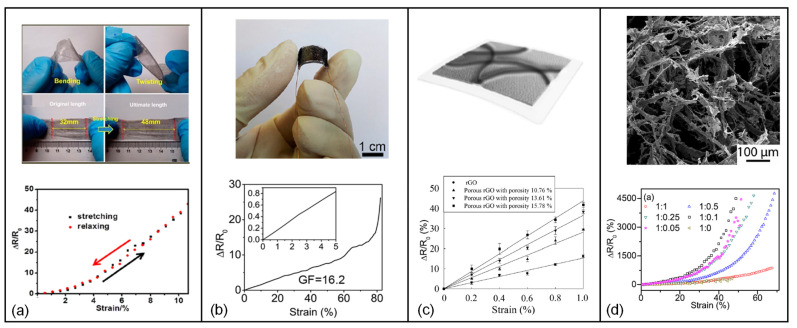
rGO graphene-based strain gauges: (**a**) digital photos of arbitrarily bent and twisted strain sensor and the relative resistance changes versus strain curves of the strain sensor under various strains. Adapted with permission from ref. [172]. Copyright (2015) American Chemical Society. (**b**) photograph of rGO strain sensor and relative resistance-strain curve of a rGO strain sensor recorded at a stretching rate of 10% min^−1^. Adapted with permission from ref. [177]. Copyright (2016) American Chemical Society. (**c**) porous rGO membrane on a PET substrate and the relative variation of resistance versus strain for rGO and porous rGO membranes with different membrane porosities, Reprinted with permission from ref. [168]. Copyright (2016) AIP Publishing. (**d**) SEM images of the cellulose-rGO composites and the relative resistance change, R/R_0_, as a function of the applied strain. Reprinted with permission from ref. [169]. Copyright (2018) Elsevier.

Stretchable and ultrasensitive strain sensors have been developed by combining the benefits of reduced graphene oxide (rGO) microtubes with elastomers. The photos of the arbitrarily bent and twisted strain sensor and the relative resistance change in the sensor versus strain curves is presented in Figure 7a. The sensors can be stretched to more than half their initial length, demonstrating long-term endurance and good selectivity to specific strain under a variety of disturbances. This sensor’s sensitivity can reach to a GF of 630 under 21.3% applied strain; more significantly, it can be readily adjusted to meet a variety of needs [172]. To obtain a higher stretchable strain gauge, a fish-scale-like graphene-sensing layer was fabricated. Fish-scale microstructure, which is shown in Figure 7b, offers the strain sensor with a large stretchability (up to 82% strain), high sensitivity (a gauge factor of 16.2–150), and high cycling stability (>5000 cycles). Incorporating this graphene-based strain sensor into a wearable system allows it to successfully detect the whole range of human movements due to tiny deformations.

Controlling the porosity of an rGO membrane has also been shown to effectively increase the gauge factor, wherein, at a membrane porosity of 15.78% and applied strain of less than 1%, the gauge factor reaches a maximum of 46.1. For applied strain of less than 1%, the gauge factor of rGO membrane has been shown to substantially rise with increasing membrane porosity (Figure 7c) [168]. Stretchable strain sensors were also created using porous cellulose-PDMS composites comprising nanocarbon materials (rGO and carbon nanofibers). Results showed that combining rGO with a small amount of carbon nanofibers (CNFs) alters the gauge factor of the composite in a manner that increases the GF from 3.4 (when there is no CNF, only cellulose-rGO) to 9.4 (when mass ratio of rGO to CNF is 1:0.1), and for larger CNF content beyond 10% to the level that rGO:CNF has 1:1 ratio the gauge factor decreases (Figure 7d) [169].

Graphene nanopapers which are three-dimensional, highly stretchable structures composed of crumpled graphene and nanocellulose are also employed in strain sensing applications. As such, stretchable nanopapers were made by vacuum filtering of free-standing flexible nanopapers, and their 3D microporous structure allows for their effective embedment in elastomer matrix. Stretchability was effectively increased from 6% for flexible nanopaper to 100% for stretchy nanopaper. Stretchy nanopaper-based high-strain sensors had a gauge factor of 7.1 at 100% strain, which is more than ten times greater than stretchable CNT and AgNW sensors [173]. In another study, polymerized rGO was decorated on electrospun thermoplastic polyurethane (TPU) mat to prepare a multifunctional strain sensor [175]. The high stretchability of the sensor (>550%) allowed it to achieve various gauge factors in different ranges of applied strain reaching a GF of 6583 at strains exceeding 140%.

## 5. Applications of Graphene-Based Strain Sensor

Strain sensors have been utilized in a broad range of applications. In the following parts, two major application areas of graphene-based strain sensors, namely, wearable devices and physical sensors (i.e., accelerometer, pressure sensor) are discussed.

### 5.1. Wearable Devices

**Figure 8:**
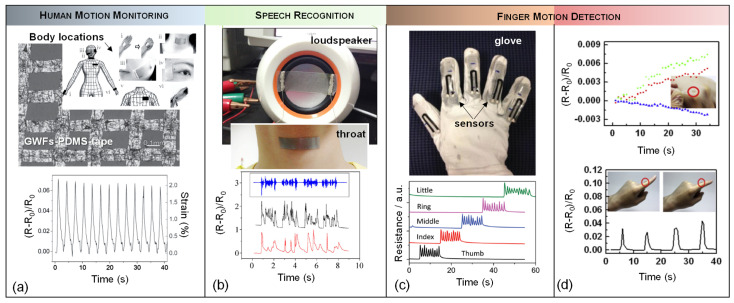
(**a**) Optical microscope image of graphene woven fabrics (GWFs)-PDMS-tape composite film (scale bar 0.1 mm) and the relative resistance change as a function of applied strain varying between 0% and 0.2%. Reprinted with permission from ref. [187]. Copyright (2014) John Wiley and Sons. (**b**) Photograph of a strain sensor attached to the vibrating membrane of a loudspeaker and to a participants’ throat, where similar change in relative resistance was observed as the same sentence was played from the loudspeaker (black curve) and read out loud (red curve). Reprinted with permission from ref. [189]. Copyright (2015) Springer Nature. (**c**) Application of graphene nanopaper-based sensors on a glove is imaged and the transitions between the corresponding resistance changes of the strain sensor by the motion of each of the fingers. Reprinted with permission from ref. [173]. Copyright (2013) John Wiley and Sons. (**d**) Observation of relative resistance changes in the strain sensor on a glove when the finger bends or unbends and using a rosette gauge on the glove to detect the direction of principal strain by applying stretch gently. Reprinted with permission from ref. [143]. Copyright (2013) Elsevier.

One major area where strain sensors are used vastly as different applications is wearable electronics. For healthcare applications, wearable sensors have been attached to gloves, organs, and skins to observe physiological activities of the body such as monitoring heart rate, wrist pulse, motion, blood pressure, intraocular pressure, vibration of vocal cords, movement of joints and other health-related situations [65,66,67,180,181,182,183,184,185]. Since the mechanical properties of piezoresistive materials such as flexibility and stretchability are vital in wearable sensors, only a limited selection of materials meet the requirements to be used in these sensors. Therefore, graphene has attained promising interest compared to other materials [186,187,188]. Inspired by this fact, a graphene woven fabric (GWF) on PDMS and a medical tape composite has been reported as a wearable strain sensor for detecting body motion (Figure 8a) [187]. The sensor offers different gauge factor values of 35, 103 and 106, at strains of 0.2%, 2–6% and >7%, respectively.

In another study, a device for detecting and identifying sound-signals with the help of strain sensing mechanism of the graphene woven fabric (GWF)-based sensor on PDMS was investigated [189]. The sensor was utilized in the form of a patch that was attached to human throat to investigate resistance changes due to the movement and vibration of throat muscles during vocalization. To benchmark the sensor response, the same sentence was played by a loudspeaker and read out loud by a person who had the sensor attached to his throat, where similar resistance change was observed in both testing conditions. The sensor showed high sensitivity (even to the low frequencies), and reliability.

Figure 8c demonstrates graphene nanopaper-based strain sensors attached onto a feather glove as a possible real-life application of wearable sensors in order to detect the movements of the fingers. The response behavior of sensors located on the fingers were tested by bending and stretching all five fingers at a frequency of 1 Hz. Also, high strain in range of 0–100% was measured in this experiment. The gauge factors for stretchable nanopaper increased from 1.6 at 10% strain to 7.1 at 100% strain. Besides, the solution process-based fabrication method made the strain gauge superior in terms of cost and mass production ability [173]. To make high-strain sensors, this technique uses crumpled graphene and nanocellulose. Free-standing flexible nanopapers were created by vacuum filtering, and their 3D structure allowed them to be successfully embedded in an elastomer matrix to produce stretchy nanopapers. However, there is still a restriction about measuring high strains over 50% that are caused by stretching and contracting motions in human joints. So, using nanopapers may be a solution to detect strain over 100%. This application shows a gauge factor of 7.1 at 100% of strain which is ~10 times higher than those of 1D materials such as CNTs and AgNWs in a similar device arrangement.

A single strain gauge is typically capable of measuring the strain that has the same direction with the position of the gauge [180]. That is why the aligning a strain sensor within the direction of the strain is necessary to obtain an immediate response to the deformation. Since recognizing the principal strain directions on human skin is generally impractical, it is not easy to measure the predominant strain directly. Figure 8d illustrates an application onto a glove as a sensor to detect the direction and magnitude of the predominant strains. Changes occurred in a normalized resistance value when the rosette gauge was stretched. When the finger bends, the strain caused by the finger is taken up by the first glove layer and then transmitted to the rosette gauge. The rosette gauge responds quickly to the bending of the finger, and its signal amplitude is proportional to the amount of strain caused by the bending: the more the finger bends, the more the signal amplitude grows. The strain caused by bending the finger is estimated to be 1.3%, 1.2%, 1.4%, and 1.6%, respectively, based on the resistance change. The strain sensor also was recovered perfectly after straightening the finger. Both the magnitude of the applied force and the direction of the major strains on the skin were detected simultaneously by setting the strain gauges in the rosette arrangement. The green, red and blue lines in the resistance plot in Figure 8d correspond to 3-gauge sensor a, b and c. The ”a” gauge and the other two gauges, ”b” and ”c”, are positioned at the same distance and are oriented at the same angle with respect to the “a” gauge [143].

Along with wearability, applications deeming stretchability and flexibility are yet another area where the use of graphene as a strain sensing element offers advantages. For instance, a highly sensitive graphene embedded viscoelastic polymer nanocomposite was reported as a flexible strain sensor, which could measure very small pressures of pulse, blood pressure and even the minute mechanical loading due to walking of small insects. In this work, the mobility of graphene fillers was enhanced by a lightly cross-linked polymer matrix providing high viscosity. The resulting nanocomposite exhibited a remarkable change in resistance as it was subjected to an applied strain. It yielded a high gauge factor of more than 500, and the temporal relaxation of electric resistance upon deformation [186].

### 5.2. Physical Sensors

Measuring and monitoring the acceleration is vital in various cases such as monitoring activity in biomedical and healthcare applications [190,191], stability control and crash detection in automotive industry [192,193,194], consumer electronics such as cellular phones [1,195], navigation systems, robotic and military applications [196,197,198]. As a result, in recent decades, MEMS accelerometers have been researched widely. By measuring the amount of deflection in a cantilever or membrane and the corresponding resistance change, the magnitude of acceleration can be estimated with piezoresistive sensors [199,200].

**Figure 9:**
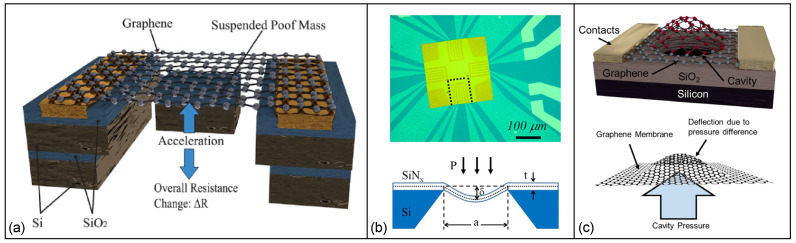
(**a**) 3D design of an accelerometer with a suspended graphene ribbons and an attached proof mass [202]. (**b**) 1-Optical microscope image of graphene piezoresistors on silicon nitride membrane and the schematic of suspended silicon nitride layer under the applied differential pressure. Reprinted with permission from ref. [23]. Copyright (2013) AIP Publishing. (**c**) Schematic of a suspended graphene cover a circular cavity to measure the chamber pressure due to pressure difference [64].

So far, several studies have reported piezoresistive-based accelerometers employing graphene [201,202,203]. For instance, a piezoresistive transducer was built by using a suspended double-layer graphene ribbon with significant built-in stress (order of 230 to 440 MPa) that shows a noticeable improvement on the static and dynamic characteristics of the device (Figure 9a) [201]. It was reported the Young’s modulus was decreased for small deflection and applied strain to the device. Moreover, the device has proof mass that is at least three orders of magnitude less than frequently reported piezoresistive silicon accelerometer proof masses that show a greater magnitude of ΔR/R per proof mass volume compared to previously reported piezoresistive accelerometers.

Likewise, graphene-based suspended, planar, spongy and double layer microstructures have been produced as highly flexible and sensitive pressure sensors. Zhu et al. [23] fabricated graphene meandering patterns on a square silicon nitride membrane. Pressure applied to the graphene membrane caused it to bend and deform into a concave shape in varying degrees. The graphene sensor’s piezoresistive effect and out-of-plane deflection allowed it to measure the applied pressures. Accordingly, a gauge factor of 1.6 for graphene and a dynamic range from 0 mbar to 700 mbar for the pressure sensor was obtained (Figure 9b).

Another suspended graphene membrane was fabricated on rectangular and circular cavities (with diameter of 24 μm and depth of 1.5 μm) etched into SiO_2_ layer where the membrane was able to deflect due to pressure differences in the sealed cavity and in the pressure chamber. A superior sensitivity in pressure sensing was observed compared to silicon and CNT-based pressure sensors. The maximum GF of the piezoresistive sensor was 4.33 with an average value of 2.92 which, unlike silicon piezoresistive sensors, was unaffected by dopant concentration or crystallographic orientation (Figure 9c) [64].

## 6. Conclusions and Outlook

Strain sensors have rapidly developed in the modern era due to the wide range of applications that they find use in, and graphene is a compelling candidate in this field. Graphene stands out as a suitable sensor material due to its remarkable physical, mechanical, and electrical properties as well as high flexibility and stretchability. Existing methods to synthesize, pattern and transfer graphene have resulted in different types of graphene with diverse electrical and mechanical properties that can be helpful towards development of ultra-sensitive and stretchable strain sensors, provided that the said properties can be accurately controlled with excellent run-to-run repeatability.

It is due to the shortcomings of accurate process control in graphene synthesis methods (including CVD, exfoliation, chemical reduction) and material to device integration challenges that cause graphene-based strain sensors to have a wide range of gauge factors which also reflect in the scattered gauge factor values reported in published literature. Essentially, using different types of graphene on different kinds of substrates calls for different fabrication schemes, and results in strain gauges with varying performance and form factors. Although huge progress has been made in developing and studying graphene-based strain sensors in recent years, it is clear that further research is necessary. Future efforts should focus on minimizing the challenges in synthesis techniques and improving transfer/integration approaches, as well as exploring the underlying mechanisms to improve device sensitivity and stability.

## Figures and Tables

**Table 1:** Classification of graphene-based strain gauges in terms of the method used to obtain graphene, along with the device topology and performance metrics like gauge factor and strain range.

Graphene Synthesis Method	Device Topology	Gauge Factor	Strain Range	Ref.
CVD	RPECVD graphene on mica substrate	325	0.30%	[140]
	Graphene-nano graphene sheets on finger	500	1%	[136]
	Suspended CVD graphene membrane	1.6	0.25%	[23]
	Suspended CVD graphene membrane	3.67	0.29%	[141]
	CVD graphene on suspended perforated SiNx membrane	4.4	0.22%	[142]
	Graphene glow sensor	2.4	1.8%	[143]
	CVD graphene woven fabric on PDMS	10^6^	10%	[144]
	Graphene-graphene woven on PDMS	223	3%	[145]
	Fragmented graphene foam on PDMS	15–29	77%	[146]
	Graphene tactile sensor	1.4	-	[147]
	CVD graphene on PDMS	6.1	1%	[148]
	braided graphene belts sensor	175.16	55%	[149]
	planar and crumpled graphene	20.1	105%	[150]
	graphene/g-C_3_N_4_ heterostructure on PDMS	1.89	25%	[151]
	Graphene-single layer graphene on finger	42.2	20%	[26]
	Graphene wrapped CNTs	20	1.20%	[152]
	PDMS graphene reinforced CNT network	0.36	-	[153]
Exfoliated graphene	Spray-deposited graphene on a flexible plastic substrate	10–100	1.70%	[154]
	Mechanical exfoliated graphene on a silicon wafer	10–15	0.08%	[155]
	Mechanical exfoliated graphene nanoribbons	0.6	0.054%	[156]
	Mechanical exfoliated graphene nanoribbons	8.8	5%	[157]
	Graphene-printed fragments	125	0.30%	[158]
	Mechanical exfoliated graphene nanoribbons	1.9	3%	[159]
	Graphene solution coated on polypropylene film	1000	0.05–0.265%	[160]
	Polymer-functionalized hydrogen-exfoliated graphene	10	0.35%	[161]
	Graphene nanoplatelet on PDMS	62.5	2.5–25%	[162]
	PDMS-graphene nanoplatelet/CNT hybrids	1000	18%	[163]
	Carbon nanotube-graphene nanoplatelet hybrid film	<1	-	[164]
rGO	Mechanical exfoliated Graphene ripple on PDMS	−2	20%	[165]
	3D graphene foam-PDMS nanocomposite	178	30%	[166]
	rGO on a PET substrate	61.5	0.01–0.04%	[167]
	rGO membrane porous structure	15.2–46.1	1%	[168]
	PDMS-cellulose-rGO/CNFs hybrids	9.4	70%	[169]
	3D porous PDMS CNT/rGO hybrid	1.6	80%	[170]
	Polyurethane-silver nanowires/graphene hybrids	20–400	0.3–1%	[171]
	rGO-microtube on PDMS	630	50%	[172]
	Crumpled graphene-nanocellulose composite on elastomer matrix	−7.1	100%	[173]
	rGO doped with polystyrene nanoparticles (PS) on PDMS	250	1.05%	[174]
	Polymerized rGO on TPU	23.15–6583	550%	[175]
	rGO mesh on an LCP substrate	375–473	0.1–1.4%	[176]
	rGO-fish scale like on an elastic tape	16	82%	[177]
	rGO-conductive cotton fabric	-	0.02–0.35%	[178]
	rGO-FET on polyethersulfone (PES)	20	50%	[179]
